# Alternate Splicing of Transcripts upon *Mycobacterium tuberculosis* Infection Impacts the Expression of Functional Protein Domains

**DOI:** 10.1002/iub.1887

**Published:** 2018-08-18

**Authors:** Haroon Kalam, Kartikeya Singh, Komal Chauhan, Mary F. Fontana, Dhiraj Kumar

**Affiliations:** Cellular Immunology Group, International Centre for Genetic Engineering and Biotechnology, Aruna Asaf Ali Marg, New Delhi, India

**Keywords:** mycobacterium, RNA-seq, alternative splicing, JAL2287, pfam, kinase domain

## Abstract

Previously, we reported that infection of human macrophages with Mycobacterium tuberculosis (*Mtb*) results in massive alterations in the pattern of RNA splicing in the host. The finding gained significance since alternate spliced variants of a same gene may have substantially different structure, function, stability, interaction partners, localization, and so forth, owing to inclusion or exclusion of specific exons. To establish a proof-of-concept; on how infection-induced RNA splicing could impact protein functions, here we used RNA-seq data from THP-1 macrophages that were infected with clinical isolate of *Mtb*. In addition to re-establishing the fact that *Mtb* infection may cause strain specific alterations in RNA splicing, we also developed a new analysis pipeline resulting in characterization of domain maps of the transcriptome postinfection. For the sake of simplicity, we restricted our analysis to all the kinases in the human genome and considered only pfam classified protein domains and checked their frequency of inclusion or exclusion due to alternate splicing across the conditions and time points. We report massive alterations in the domain architecture of most regulated proteins across the entire kinases highlighting the physiological importance of such an understanding. This study paves way for more detailed analysis of different functional classes of proteins and perturbations to their domain architecture as a consequence of mycobacterial infections. Such analysis would yield unprecedented depth to our understanding of host-pathogen interaction and allow in a more systematic manner targeting of host pathways for controlling the infections.

## Introduction

Tuberculosis (TB), caused by *Mycobacterium tuberculosis (Mtb)* continues to constitute a significant fraction of population suffering from infectious diseases globally. Despite having very effective treatment regimen available, this pandemic is far from getting over due to factors like emergence of drug resistant strains and co-morbidities like HIV, aging, and malnutrition (1–4). At least one of the reasons for emergence of drugresistance in TB is that so far we continue to follow one-sizefits-all concept. In the field, *Mtb* isolates are known to display wide diversity in their growth patterns, gene expression patterns, metabolism etc. and therefore may show diversity in their susceptibility to standard-of-care drugs (5–13). In part, due to factors mentioned above, there has been a surge in efforts to evolve alternate strategies of targeting *Mtb* like hostdirected approaches. We previously identified a large number of host genes, which were important for intracellular survival of *Mtb* as well as several diverse clinical isolates within macrophages (7, 14). The fundamental premise is that—if we understand the host factors, which play an important role in intracellular mycobacterial survival—they can be targeted to achieve bacterial killing irrespective of the drug resistant profile of the pathogen. Several studies in the past have successfully identified such host targets and showed its ability to target both drug sensitive and drug resistant strains of *Mtb* (7, 14–19).

One of the major host response machinery that gets dramatically altered upon mycobacterial infections is gene expression. This includes immediate early changes in gene expression as well as long-term shaping of responses by persistent transcriptional reprogramming (9, 20–24). In a recent study we showed that the impact of mycobacterial infection on host transcriptional machinery is much deeper than understood and that in infected macrophages, patterns of host RNA splicing was globally perturbed. More importantly, we showed, that splicing events were specifically directed towards genes/molecules, which could play an important role in deciding the host response to the bacterial infection (25). Alternate splicing of transcripts could have massive impact on the cellular physiology since different spliced variants of a given gene could differ in their structure, function, stability, interaction pattern and localization (25, 26). However, there is no systematic study to establish the magnitude of infection induced alternate splicing on the functioning of final protein products. Another layer of complexity is brought in by large variations in the field strains of *Mtb* (27–29), which could also vary in the magnitude of perturbations of host RNA splicing upon infection. In a proteomic study previously, it was reported that clinical, drug-resistant variants of *Mtb* show markedly different protein expression profile compared to the laboratory strain H37Rv (30).

In view of the above, in this study, we have used a clinical isolate of *Mtb*, which is also a multi-drug resistant (MDR) strain to identify alterations in host RNA splicing patterns upon infection in THP-1 macrophages, which was compared with our previous studies where laboratory strains H37Rv and H37Ra were used for similar studies. We developed a new analysis pipeline to perform a domain-level mapping of host kinome as a consequence of infection-induced RNA splicing. We show that infection induced RNA splicing has substantial impact on domain representations across the expressed transcripts. This study therefore highlights newer challenges to developing alternate host-directed therapeutic strategies.

## Results

### Transcriptome Profile of Avirulent, Virulent or Clinical Isolate of Mtb Infected Macrophages

**FIG 1:**
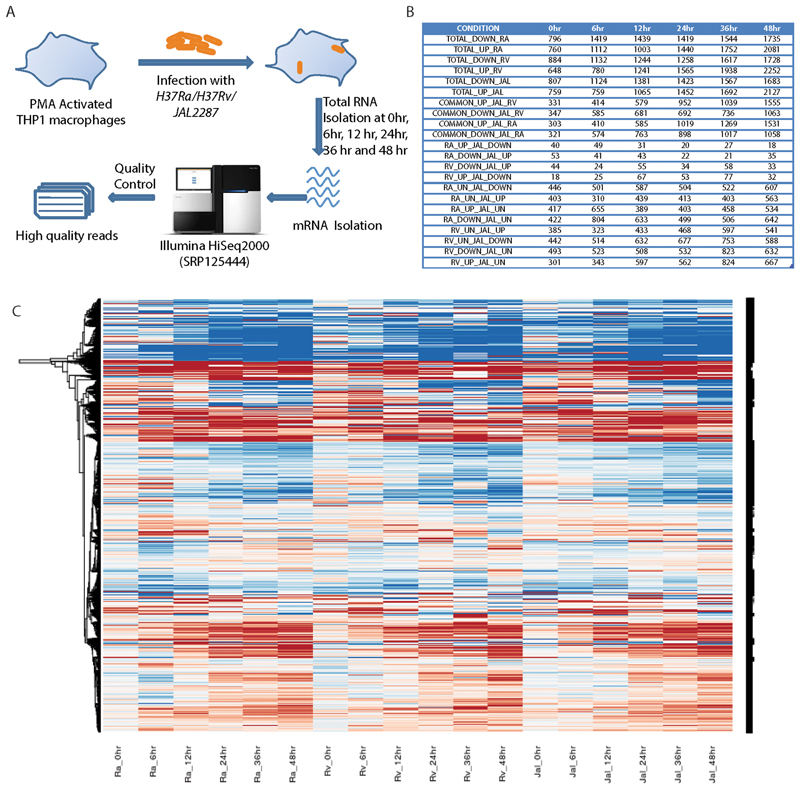
RNA-seq analysis of THP-1 macrophages infected with avirulent, virulent or clinical isolate of *Mycobacterium tuberculosis*. A. Flow-chart of the RNA-seq experiment. THP-1 macrophages were infected with H37Ra, H37Rv, or JAL2287 for different time points, total host RNA was isolated and processed for RNA-sequencing.B. Comparative table for differentially expressed genes between H37Ra, H37Rv, and JAL2287 infected macrophages at gene level quantification (up: two fold increase in expression; down: two fold decrease in expression; UN: no change in expression)C. Global gene expression profile of THP-1 macrophage cells upon infection with H37Ra, H37Rv, or JAL2287 at 0, 6, 12, 24, 36, and 48 h post infection.

THP-1 macrophages were infected with the clinical isolate (JAL2287) of *Mtb* following the protocols as reported earlier for avirulent (H37Ra) and virulent (H37Rv) *Mtb* infections (experimental set-up schematically shown in Fig. 1A) (25, 30). For each sample approximately 180 million paired end reads were aligned to human reference genome build hg19 using splicing aware Tophat aligner (31). More than 75% of reads aligned to hg19 in each sample (Supporting Information Table S2). Out of the aligned reads ∼94% aligned to genes, ∼3% aligned to intronic region and ∼2.5% aligned to intergenic region. Transcriptome reconstruction was done using cufflink package (32). Absolute quantification at both gene and transcript level was performed using FPKM (Fragments Per Kilobase of transcript per Million mapped reads) units. Differentially regulated genes and transcripts compared to uninfected control were identified using cuffdiff package using negative binomial distribution. Quality control of all aligned files was checked using BAMQC tool (33). Dispersion analysis and median expression confirmed that there was no bias in alignment. We compared the gene expression in JAL2287 infected cells with respect to H37Ra and H37Rv infected cells as reported earlier (25). The comparative expression table is shown in Fig. 1B. Overall, the number of regulated genes increased with time in all samples. We found more genes commonly regulated between JAL2287 and either H37Ra or H37Rv (row 7–10, Fig. 1B). Very few genes showed exactly contrasting expression pattern between JAL2287 and either H37Ra or H37Rv (row 11–14, Fig. 1B). This trend was also reflected in the heatmap (Fig. 1C) generated from gene expression table where most of the clusters were common across the samples. We found time point specific contrasting clusters but no global contrasting cluster emerged, suggesting most of the differentially regulated genes were commonly regulated across the strains.

### Functional Analysis of Differentially Regulated Genes

**FIG 2:**
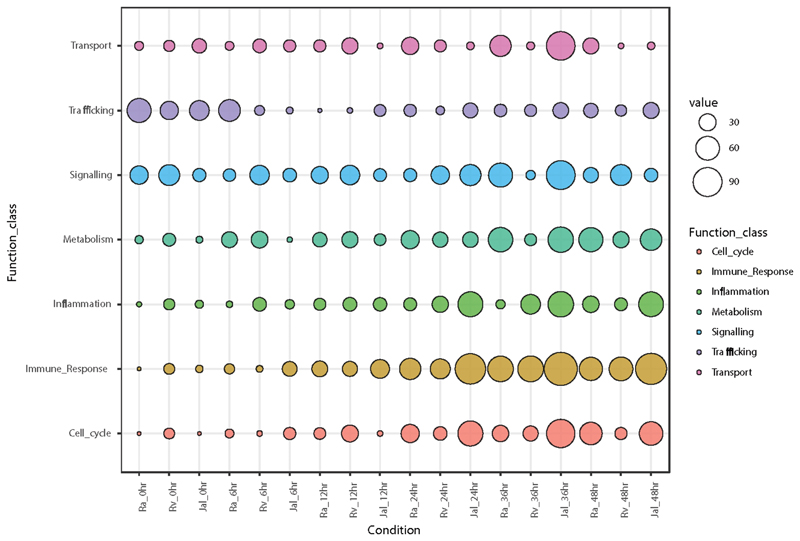
Gene Ontology enrichment analysis of differentially expressed genes in H37Ra, H37Rv, or JAL2287 infected THP-1 macrophages. Significantly enriched gene ontology classes (*P*-value < 0.001) detected at all-time points were manually classified into seven categories: cell cycle, trafficking, inflammation, metabolism, signaling, immune response and transport. Color of the circle represents a particular class and size represents the cumulative enrichment score.

Using differentially regulated genes as targets and nondifferentially regulated genes as background we performed the functional enrichment analysis and identified significantly overrepresented GO (gene ontology) terms for each sample(34, 35). Gene ontology analysis allows one to test whether the expression data is representative of the study. This can be achieved by looking at the most enriched functional classes and their relevance to the experimental conditions (14, 25). To simplify our understanding we manually classified genes into seven different ontological categories, which were also among the most enriched one in our analysis: cell cycle, trafficking, inflammation, metabolism, signaling, immune response, and transport. These ontologies have previously been shown to be associated with host-pathogen interaction in siRNA screens and microarray experiments (14, 36, 37). Moreover, to understand the extent of variation due to different infecting strains, we have also incorporated data sets from H37Ra and H37Rv infected cells, as reported previously by us (25). The enrichment of these GO terms with time and strain of infection is shown in Fig. 2. A quick look at the pattern of GO enrichment provided peeks into cellular response machinery to infections. Thus, trafficking was the one class which showed maximum regulation at early time points across all three strains, although it persisted for little longer in the case of H37Ra (Fig. 2). Genes related to cell signaling showed nearly uniform level of perturbation throughout the course of infection albeit with subtle time point specific and strain specific variations. Most other functional classes like transport, metabolism, inflammation, immune responses and cell cycle showed time dependent increase in regulation. Interestingly, most of the functional classes in JAL2287 infected cells showed highest level of regulation except transport class where JAL2287 infected cells showed minimum regulation (Fig. 2).

### Transcript Level Profile and Alternate Splicing Landscape of Infected Macrophages

**FIG 3:**
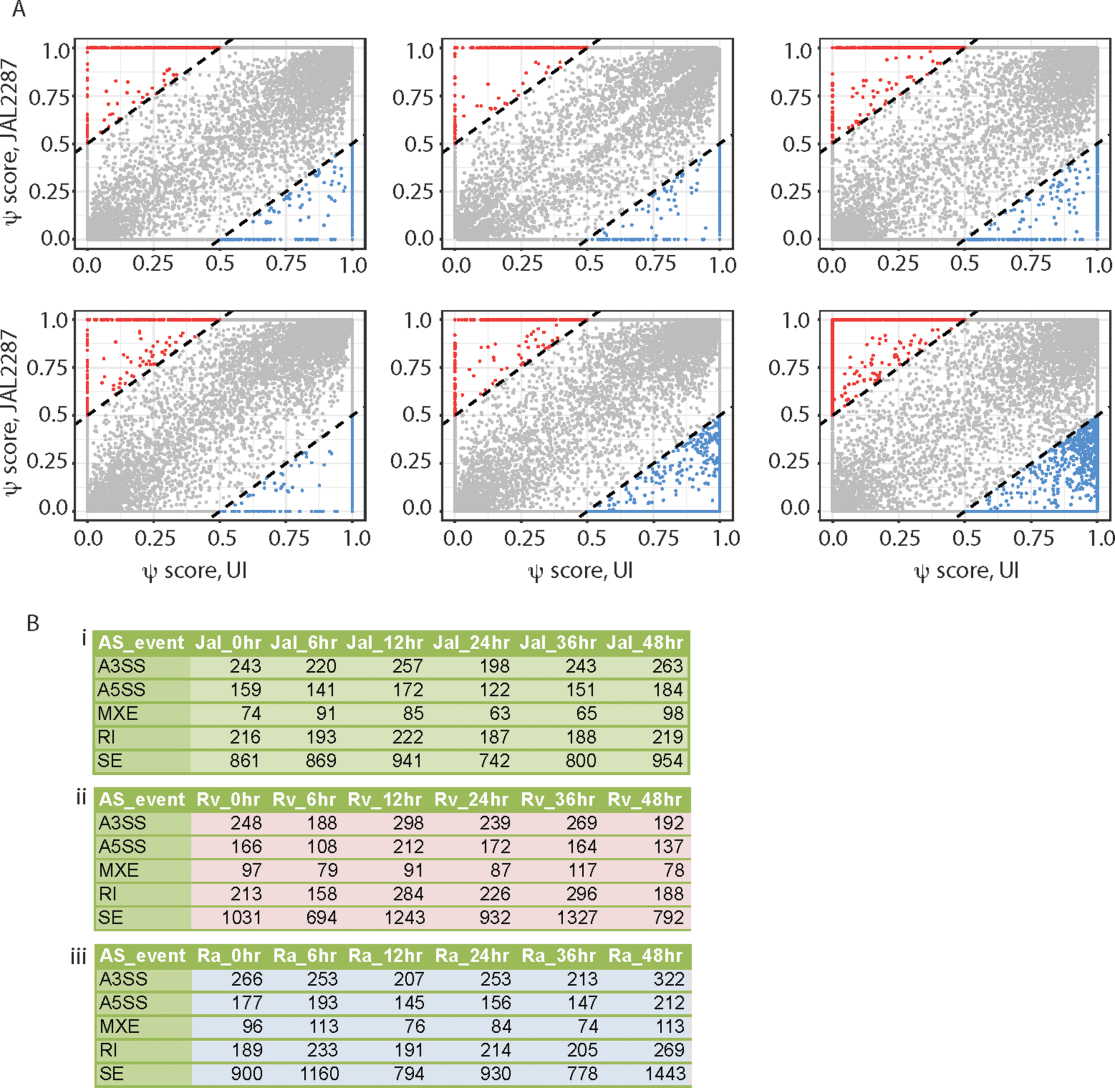
Estimation of alternate splicing in JAL2287 infected THP-1 macrophages.A. Dot plots for isoform specific psi scores between JAL2287 infected macrophage versus uninfected control plotted for each time point. Each dot represents a single transcript. The dotted lines mark the regions beyond which transcripts had higher psi-score in JAL2287 infected cells by 0.5 or more with respect to uninfected cells (red) or in uninfected cells by 0.5 or more with respect to JAL2287 infected cells (blue). B. Table showing number of significant alternate splicing events where psi-score compared to the uninfected control was higher than 0.5 across (i) JAL2287 (ii) H37Rv, (iii) H37Ra across all time points is shown here. Numbers for H37Rv and H37Ra are reproduced from Kalam et al (25) for comparative analysis. A3SS: alternate 30 splice site, A5SS: alternate 50 splice site, MXE: mutually exclusive exons, RI: retained introns, SE: skipped exons

Most of the human genes have multiple isoforms and almost 95% of genes undergo alternate splicing (38). We recently showed that transcript level expression may vary significantly from corresponding gene level expression under infection (25). Using Tuxedo pipeline we quantified the transcript level expression for all the samples of JAL2287 infected cells across the time points. Heatmap representation of isoform level expression (Supporting Information Fig. S1) was grossly different from that of gene level expression, confirming contrasting difference between gene and isoform level expression. For majority of the cases isoform expression showed larger variation at the later time points. Post-transcriptional regulation like alternate splicing can be a major reason for such variation in the isoform expression. Alternate splicing has been classified in five major events: Exon skipping (SE), mutually exclusive exons (MXE), retained intron (RI), alternative 30 splice site (A3SS) and alternative 50 splice site (A5SS). To get a statistically qualified estimation of alternate splicing we followed SUPPA computational pipeline (39). Inclusion levels were determined by generating alternate splicing event list from the GTF (Gene Transfer Format) file and modeled over transcript abundance estimation. Differential alternate splicing in a particular infected sample compared to uninfected sample was determined by taking a very stringent cut-off of 0.5 by SUPPA. Switch like events (delta Percent Spliced In score 1 and −1) were observed where an exon is completely spliced out at a time point under infection. Across the time points under JAL2287 infection psi score of uninfected sample (X-axis) was plotted against psi score of infected sample (Y-axis) (Fig. 3A). We observed, with time, significant differential alternate splicing, specifically switch-like events increased under JAL2287 infection compared to uninfected, as may be noted by a large number of red dotes aligned to Y-axis (psi-score of UI close to zero in uninfected) or X-axis (psi-score of 1 in JAL2287 infected cells, while less than 0.5 in UI (Fig. 3A). At 48 h postinfection we found maximum number of switch like events. We applied an unsupervised clustering strategy t-distributed stochastic neighbor-embedding algorithm on the psi score table and found seven clusters (Supporting Information Fig. S2). Each clusters corresponds to a type of alternate splicing class. There are only five types of alternate splicing events that are well characterized and have been quantified using RNA-seq. Quantification of complex alternate splicing event and overlapping events is very challenging and needs further investigation. For the five well classified classes of alternate splicing events, we counted the number of each of the events across the time points in JAL2287 infected cells as shown in Fig. 3B. These numbers were also compared with the corresponding numbers in H37Ra and H37Rv infected conditions (25). JAL2287 infected cells overall did not show any outstanding pattern of AS events with respect to what was noted in the case of H37Ra and H37Rv infected cells (Fig. 3B).

### Isoform Features and Alternate Splicing upon *Mycobacterium tuberculosis* Infection

**FIG 4:**
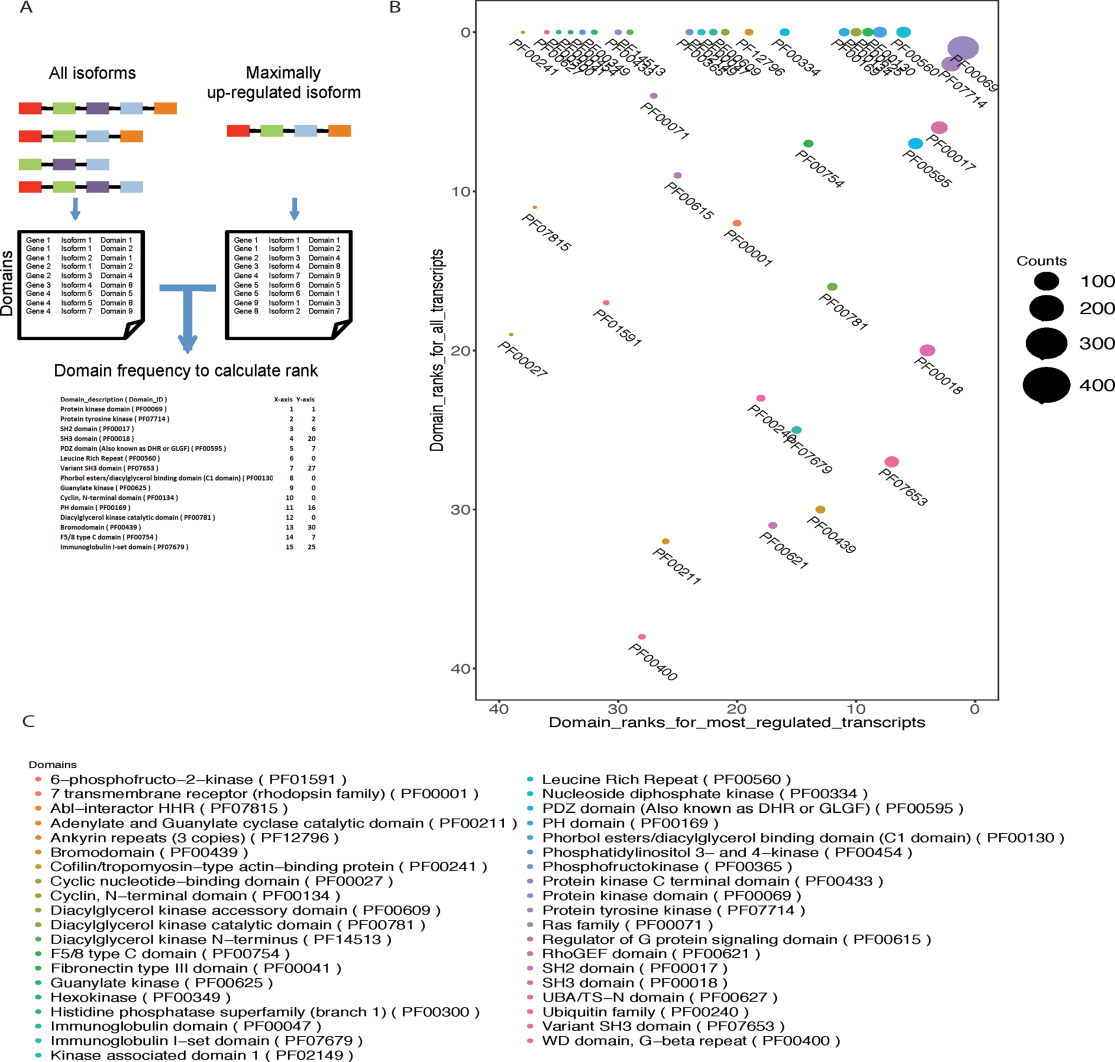
Comparative domain distribution between maximally upregulated versus all isoform in JAL2287 infected THP-1 macrophage. A. Using interpro signatures domains were identified for each transcript. Frequency of pfam domains was calculated in maximally upregulated isoform per gene and compared with pfam domain frequency when all the expressed isoforms were considered. In all isoform condition a single gene will have multiple isoform while in maximally upregulated case there is only one isoform per gene. These domains frequency were used to calculate the rank in respective class as shown here.B. Dot plot of rank of domains in all isoform (Y-axis) versus rank in maximally upregulated isoform (X-axis) case. The size of the dot represents the frequency of that domain in JAL2287 infected sample 48hr post infection.C. List of domains represented in Fig. 4B.

We next wanted to understand how changes in splicing pattern could impact on the cellular functions. In human many isoforms per gene have been catalogued as predicted transcript sequences and most of them are functionally uncharacterized. With GTF file generated after transcript reassembly, we mapped all the isoforms detected in the reassembly and chose the longest open reading frame (ORF) for each transcript using TransDecoder pipeline (40). In brief, coding regions were identified by minimum length open reading frame and a log likelihood scoring estimation per transcript, which was further subjected to position specific scoring matrix (PSSM) to identify the correct start codon prediction. These ORF were translated into peptides and used for domain search and identification using all 14 Interproscan signatures (41). This analysis resulted in a dataset of more than 1 million data points. Analyzing the entire dataset thus generated was very complex and therefore for further downstream analysis we took only those domains identified per transcript in pfam database. Moreover, as a proof-of-concept, to understand the domain level implication of alternate splicing, we performed further downstream analysis only on kinases. The motivation behind selecting kinases was that they are among the most important molecules when it comes to signal transduction, a key mechanism that gets perturbed upon mycobacterial infections. Moreover, kinases are mostly multidomain proteins where individual domains are known to influence enzymatic activity, localization, interactions, stability etc. Several host kinases in the past have been linked with intracellular mycobacterial survival including Src, Abl, PI3K, and so on (14, 42). The entire list of kinases was subclassified into two categories (i) most regulated and (ii) all the transcript expressed. Domains in each group were ranked based on their abundance (Fig. 4A). Rank of domains in most regulated at 48 hours post JAL2287 infected cells versus rank in all isoform was plotted as shown in Fig. 4B. We also calculated the frequency of each domain, which is reflected as the size of the dots in Fig. 4B. To our surprise domains like SH3 domain (PF0018), variant SH3 domain (PF07653), PH domain (PF00169), and bromodomain (PF00439), which were low on rank in all isoform, were very high when we considered only the maximally regulated isoforms. Interestingly only 10% of the entire kinase domain repertoire was represented by the domains expressed through the most regulated transcripts (Supporting Information Table S3). Similarly, several most regulated transcript lacked protein interaction domains like SH2, SH3, PH domains etc. highlighting the fact that alternate splicing can dramatically shape protein interaction patterns and thereby their downstream function and physiological consequences (Fig. 4B and C). Interestingly when compared with H37Ra and H37Rv infected cells, we could observe strain specific ranking of domains represented by the most dominantly regulated transcripts (Supporting Information Fig. S3).

## Discussion

Macrophage physiology is known to get dramatically perturbed upon *Mycobacterium tuberculosis* infection (7, 25). The perturbation mostly defines host responses to the infection and is directed towards the goal of eliminating the pathogen. The most specific response signature arises in terms of changes in gene expression, which not only decides the immediate fate of infections but also shapes the long-term response of host against the infection. In the past changes in gene expression were used as the hallmark of cellular response to infections. Moreover, these were also extrapolated to understand how changes in gene expression impacts and shapes the host response to infections. However, recent understanding that not only gene transcription but also post-transcriptional regulatory events like RNA splicing and polyadenylation could get altered upon mycobacterial infections significantly complicates the simplistic approaches followed earlier (25). Thus it was shown that a key phagosome trafficking gene RAB8B, during virulent infections, gets spliced in a way that results in synthesis of truncated transcript, which could not form a functional protein (25). Studies in the past report how exclusion or inclusion of specific exons as a consequence of splicing impacts the functional property of a given protein and in turn the physiology (26). Several examples of alternate splicing are reported which are developmental stage specific and contribute to the requisite function. For example, IL33 and ST2 were reported to regulate *UCP1* splicing in thermogenic adepocytes which helps during transition from in utero life to post-natal life (43). In cancer, gain or loss of specific exons have been linked to gain or loss of function of target proteins resulting in transformation (44). Thus it is reported that an alternate spliced variant of ATF2 can drive melanomas (45). Similarly, loss of exon 2 from NFE2L2 by alternate splicing can lead to a loss of protein domain and thereby interaction with KEAP1 resulting in activation of oncogenic pathways (46). While several systematic studies are available addressing the role and impact of alternate splicing in the pathogenesis of cancer, there is very limited literature on how induced alternate splicing, like those observed during infection of macrophages with *Mtb* could impact the domain architecture and thereby cellular immune responses.

In this study, using RNA-seq data from macrophages infected with a clinical isolate of *Mtb*, which was also an MDR strain, recapitulated our earlier observation that RNA splicing of host gets altered upon *Mtb* infection (25). A large number of expression and splicing events observed in JAL2287 infected macrophages were also similar to those observed during H37Ra or H37Rv infected macrophages suggesting there are infection specific events. Moreover, quite a few events were also unique to each of these strains, highlighting the strain-specific responses from the host. Our curiosity to understand how strain-specific variations in host RNA splicing could alter cellular response machinery led us to develop the analysis pipeline where we scanned for the presence or absence of specific protein domains in different spliced variants expressed during infection and compared with the corresponding domains when only the most regulated transcripts for each of the genes were considered. The idea here was to explore whether via changing the most dominant transcript of a given gene, could we extrapolate possible impact on the host physiology. A domain expression analysis for the entire human genome resulted in more than a million data points. To explore the utility of domain level characterization, as a proof-of-concept, we restricted our analysis to human kinases and corresponding protein domains identified through pfam. Significance of host protein kinases in host signaling and intracellular survival of *Mtb* is well established with specific details available of Src, Abl, TBK1 and so on (14, 42). As it turned out, only 10% of the entire kinase domain repertoire (including serine/threonine and tyrosine kinase domains) were represented by the most dominantly regulated transcripts. Moreover, other specific domains like SH2 domain (for binding with phospho-tyrosine residues), SH3 domains (binding with—PXXP—motifs) and several other domains for protein-protein interaction or function were variably represented due to alternate splicing.

Host-directed therapy against pathogenic diseases including tuberculosis has received significant attention in the past decade (47). One of the major arguments favoring such a measure is that unlike in the case of antibiotics, these drugs do not target pathogen directly and therefore should be effective against existing drug resistant infections. Results in this study however brings forward the intriguing concept that ‘host-directed-therapy’ against tuberculosis, which is expected to be effective irrespective of drug-resistance profile of the infecting pathogen (48, 49), may still show variability in the outcomes of treatment due to following reasons: (i) differences in the infecting strains, which could potentially vary in their potential secreted proteins profile, different strains could target different host proteins with varying degree; (ii) since several alternate splicing events are unique to infecting strains, it is likely that many key host-pathogen interaction partners are not utilized across different infections and finally; (iii) different individuals differ in their alternate splicing response upon mycobacterial infection, again impacting the host-pathogen interaction partners critical for bacterial survival. Current study therefore constitutes potential first step towards more high resolution mapping of host-pathogen interaction events, keeping in view overall changes in splicing pattern, gene expression and secretory machinery of the infecting strain, together resulting into developing potentially highly personalized treatment regimens against tuberculosis. However, delineating all these events and their respective role during mycobacterial infections is expected to be a massive challenge and would require much more concerted effort in future. Interestingly, similar crosstalk between host and the pathogen at RNA splicing level for other bacterial and viral pathogens are not yet explored, thereby unraveling a significant potential for expanding the scope of impact RNA splicing could have across various infection condition.

In conclusion, we report here massive alteration in the repertoire of protein domains expressed as a consequence of mycobacterial infection induced changes in the host transcriptome, which differs between different strains of the infecting pathogen. A more systematic and multi-disciplinary approach will be required to establish the entire part-list and their dynamics, which regulate host responses to mycobacterial infections.

## Methods

### Bacterial Strain, Cell Culture, and Infection

Human monocytic cell line THP-1 was cultured and maintained in RPMI 1640 supplemented with 10% FBS (Gibco). The THP-1 cells were differentiated into macrophages by treating them with 32 nM PMA.These were then infected by JAL2287 strain at 1:10 multiplicity of infection (M.O.I.) for 4 h followed by 2 h treatment with 200 μg/ml Amikacin to clear extracellular bacteria. The cells were then washed and kept in RPMI 1640 with 10% FBS. The media was replaced every 24 h.

### Total RNA Isolation

The RNA was isolated at 0, 6, 12, 24, 36, and 48 h from JAL2287 infected THP1 cells using MDI RNA Miniprep kit (MTRK250) according to manufacturer’s guidelines.

### Sequencing and Quality Control

Total RNA was isolated post 0, 6, 12, 24, 36, and 48 h of infection (single samples per time point) and cDNA libraries were prepared followed by paired end 101bp sequencing using Illumina HiSeq 2000 technology. Quality control was performed using FASTQC kit and reads with Phred score less than Q30 were trimmed. More than 85% of the reads passed the Q30 filter (Supporting Information Table S1) and were considered for downstream analysis.

### RNASeq Read Alignment

Human genome build hg19 was downloaded from Ensemble (http://asia.ensembl.org). Paired end RNA seq reads from indicated time points were mapped against hg19 using tophat version 2.0.9 (http://tophat.cbcb.umd.edu) with following options ‘-p 24 -G Human_ENSEMBL_coding.gtf’ where Human_ENSEMBL_Coding.gtf contains the Ensemble coding transcripts in GTF file format. No novel junctions or novel insertion-deletion were taken in account by passing the parameter ‘-no-noveljunc’ and ‘-no-novel-indel’ respectively.

### Transcriptome Reassembly and Quantification

The alignment files from tophat were assembled to create a single merged transcriptome annotation using cufflinks and cuffmerge. Gene and isoform level expression were calculated by using isoform expression method by running cuffdiff (http://cufflinks.cbcb.umd.edu/) on the merged transcriptome assembly along with the BAM files from TopHat for each sample.

### Alternate Splicing Quantification

For alternate splicing quantification we applied SUPPA algorithm (https://github.com/comprna/SUPPA). Known alternate splicing events were generated from GTF file using ‘genarateEvents’ command. Psi score per exon was calculated and then modelled to isoform level using ‘psiPerIsoform’ command for each sample. Differentially regulated AS event were identified by ‘diffSplice’ and cutoff of 0.5 was filtered.

### ORF Identification and Domain Characterization

Using in-house scripts all the transcripts were classified into two groups, most up regulated per gene and all expressed per gene. From the reassembled transcriptome GTF file all the ORF were extracted using ‘TransDecoder.LongOrfs’ command from TransDecoder package (https://github.com/TransDecoder). Further correct reading frame were determined using a loglikelihood method. Finally, the start codon were refined using a PSSM matrix. These ORF were translated to make the final peptides. Using all the 14 interpro signatures in interproscan (https://www.ebi.ac.uk/interpro/) all the domains were identified and frequency of each domain per signature was calculated. The list was reduced to only kinases for further analysis.

## Supplementary Material

Additional Supporting Information may be found in the online version of this article.

Supporting Information Figure S2

Supporting Information Figure S1

Supporting Information Figure S3

Supporting Information Table S3

Supporting Information Table S2

Supporting Information Table S1

## List of abbreviations

A3SS: Alternative 3′ splice site
A5SS: Alternative 5′ splice site
ATF2: Activating transcription factor 2
ES: Exon skipping
FPKM: Fragments per kilobase of transcripts per million mapped reads
GO: Gene ontology
GTF: Gene transfer format
KEAP1: Kelch like ECH associated protein 1
MDR: Multidrug resistant
MXE: Mutually exclusive exons
NFE2L2: Nuclear factor erythroid 2 like 2
PH domain: Pleckstrin homology domain
PI3K: Phosphatidylinositol-4,5-bisphosphate 3-kinase
PMA: Phorbol 12-myristate 13-acetate
psi: Percent Spliced In
PSSM: Position specific scoring matrix
RI: Retained intron
SH3 domain: SRC Homology 3 domain
siRNA: Small interfering RNA
ST2: Suppression of tumorigenicity 2
TBK1: Tank binding kinase
tSNE: t-distributed stochastic neighbor-embedding
UCP1: Uncoupling protein 1
