# Supplementary figures and images for: Alternate Splicing of Transcripts upon Mycobacterium tuberculosis Infection Impacts the Expression of Functional Protein Domains
Source: IUBMB Life. Author manuscript; Available in PMC 2020 Aug 22. (PMC7115969; doi:10.1002/iub.1887)

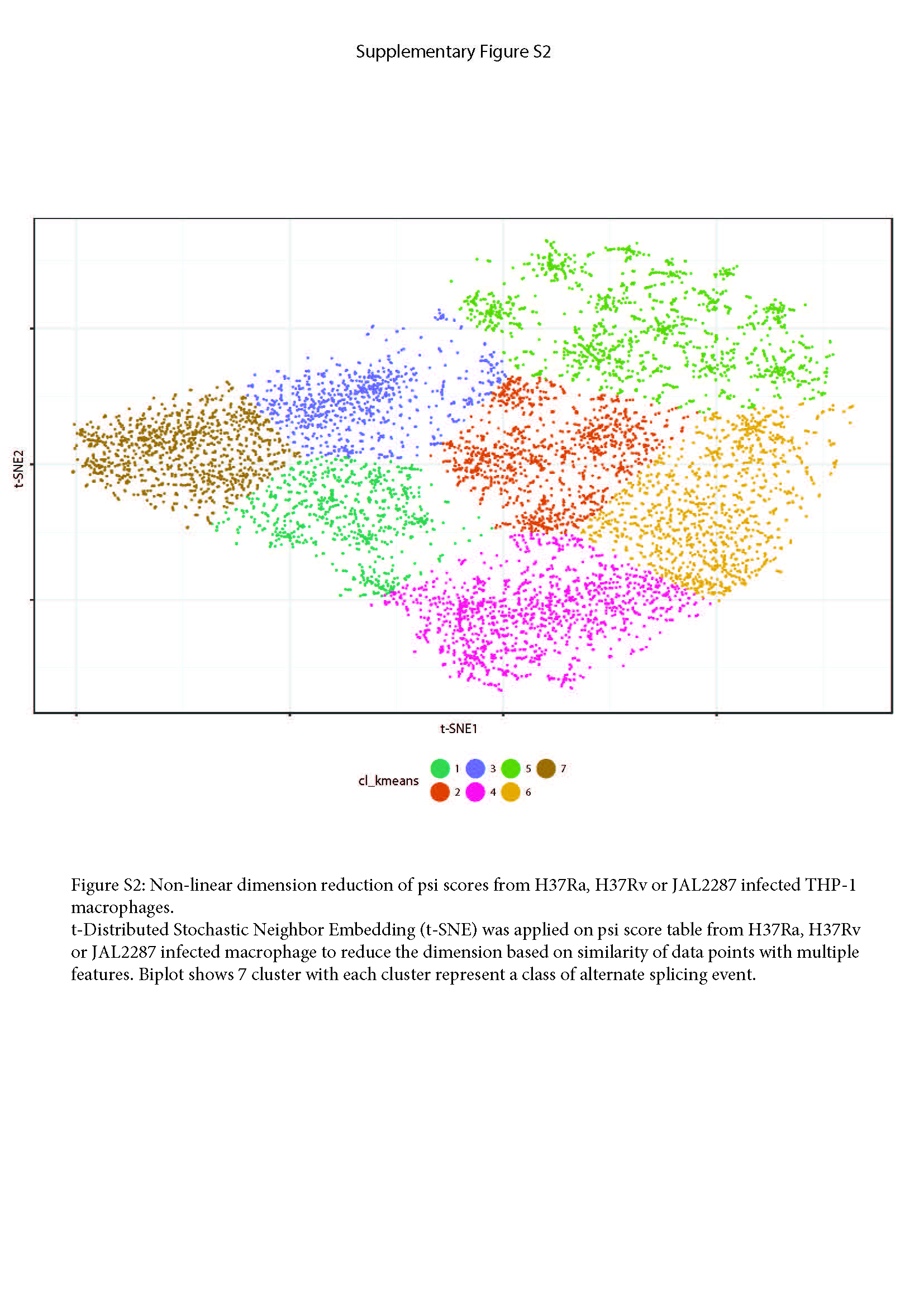

Supplement: Supporting Information Figure S2 [file EMS89260-supplement-Supporting_Information_Figure_S2.tif]

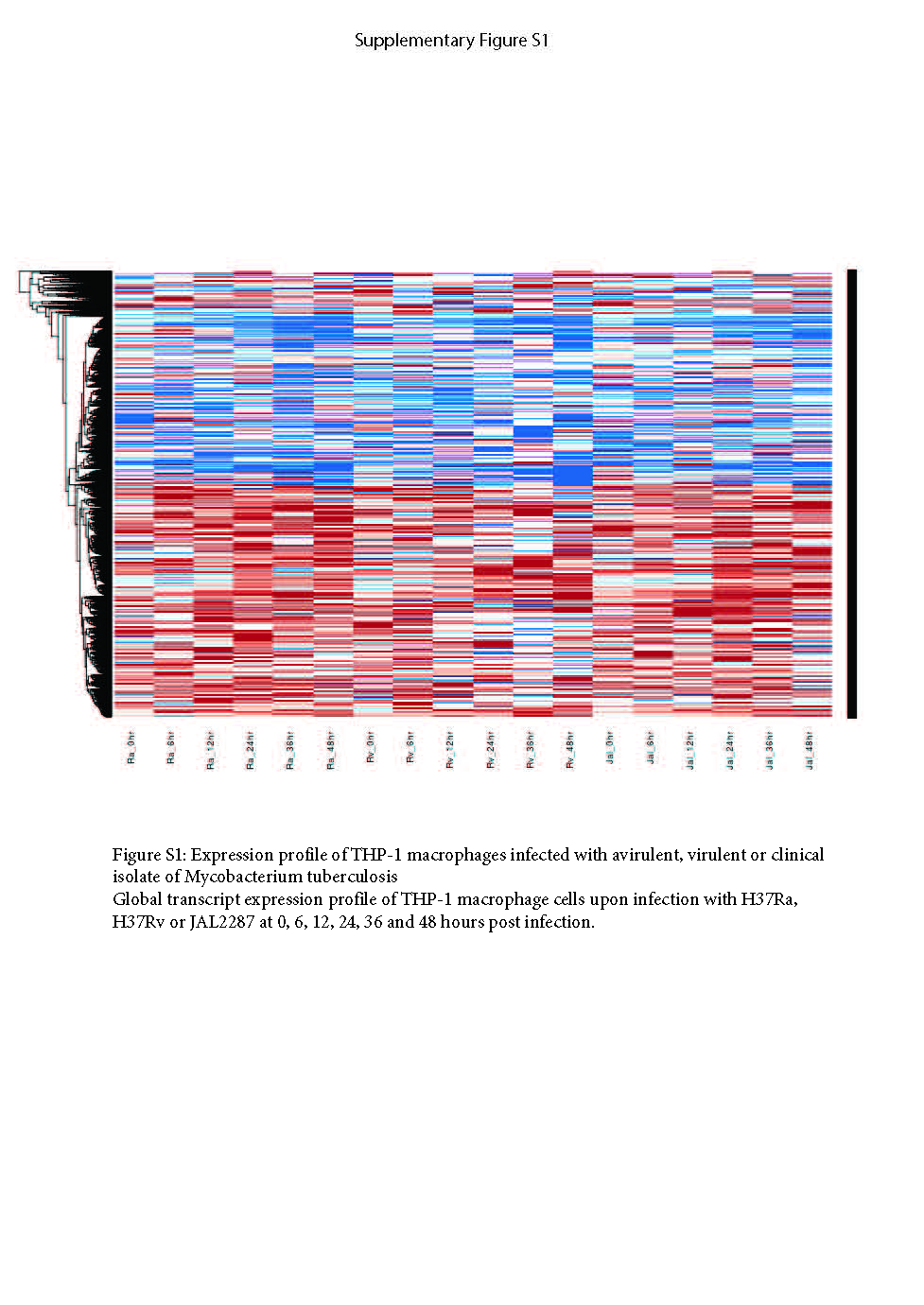

Supplement: Supporting Information Figure S1 [file EMS89260-supplement-Supporting_Information_Figure_S1.tif]

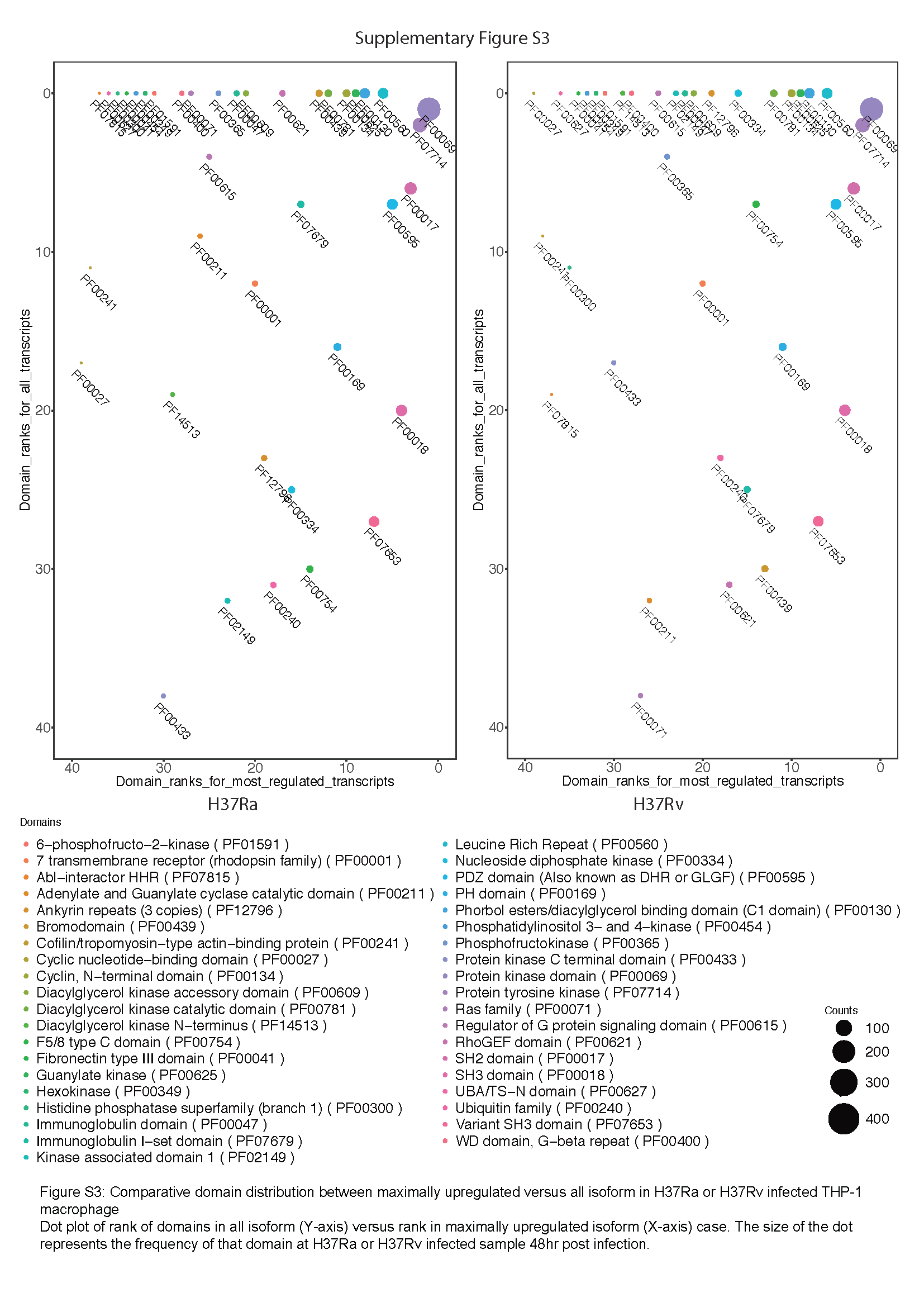

Supplement: Supporting Information Figure S3 [file EMS89260-supplement-Supporting_Information_Figure_S3.tiff]
